# 

**Published:** 1875-12

**Authors:** 


### Table of Contents.

### ORIGINAL COMMUNICATIONS.

State Board of Health—Report of Special Committee Upon the Most Effectual Means of Preventing Small-Pox in Georgia .............. 513

Remarks on the Curability of Inflammation. *By L. A. Dugas, M.D., of Augusta* .............. 526

Dry Cupping. *By B. H. Washington, M.D.* .............. 531

Description of a New Pocket Case for Physicians. *By William A. Greene, M.D., of Americus, Georgia* .............. 534

Viburnum, or Black Haw. *By John J. Addy, of Meriwether county, Ga.* 538

On the Causes, Nature, Pathology and Treatment of a Comparatively New Disease—Malarial Hematuria. *By Wm. N. Bruce, M.D.* .... 539

Atlanta Academy of Medicine .............. 551

### OUR EXCHANGES.

Dust a Cause of Catarrh .............. 558

On Hypodermic Injection of Ergotine in Certain Cases of Acute Mania. 559

Treatment of Functional Headache .............. 561

Tracheotomy in Croup and Diphtheria .............. 562

Phosphide of Zinc .............. 564

Catching Cold .............. 565

The causes of Typhoid Fever .............. 565

Large Pleuritic Effusions in Phthisis .............. 566

Treatment of Hysterical Attacks .............. 566

A Solution of Salicylic Acid .............. 567

Treatment of Ozœna .............. 567

Preservation of Morphia Solutions .............. 567

On Medical Evidence .............. 568

Jaborandi .............. 568

The Sunflower as a Preventive of Miasma .............. 568

Gall Stones .............. 569

Sleeplessness .............. 569

Gelsiminum Sempervirens in Neuralgias .............. 570

Extra Fees .............. 570

The Frontier Doctor .............. 571

### BIBLIOGRAPHICAL.

Transactions of the Medical Association of Pennsylvania .............. 571

Pamphlets ..............

### EDITORIAL.

A Rolling Stone Gathers no Moss .............. 572

Homœopathy in the University of Michigan .............. 574

Quacks .............. 576

